# Roles of the antioxidant properties of icariin and its phosphorylated derivative in the protection against duck virus hepatitis

**DOI:** 10.1186/s12917-014-0226-3

**Published:** 2014-09-24

**Authors:** Wen Xiong, Yun Chen, Yu Wang, Jiaguo Liu

**Affiliations:** Institute of Traditional Chinese Veterinary Medicine, College of Veterinary Medicine, Nanjing Agricultural University, Nanjing, 210095 China; BGI-Shenzhen, Shenzhen, 518083 China

**Keywords:** Duck virus hepatitis, Icariin, Phosphorylated modification, Antioxidant, Hepatoprotective

## Abstract

**Background:**

Duck viral hepatitis (DVH) is an acute disease of young ducklings with few convenient and effective veterinary drugs to treat. In pathology, present study mainly focused on the immune mechanism, but very few studies have concerned with the role of oxidative stress in the pathogenesis of DVH. To study the antioxidative and hepatoprotective effects of icariin and its phosphorylated derivative against DVH, we prepared phosphorylated icariin (p-icariin) using the sodium trimetaphosphate–sodium tripolyphosphate method. Ducklings were drunk with icariin and p-icariin after being challenged with duck hepatitis virus 1 (DHV-1). We recorded the number of dead ducklings, gross pathological changes in the liver, and changes in indices of oxidative stress and liver injury. The correlations between these indices were also analyzed.

**Results:**

Exposure to DHV-1 induced significant oxidative damage in ducklings. Administration of icariin or p-icariin attenuated liver pathological injury and significantly increased the survival rate, with better outcomes in ducklings treated with p-icariin than in those treated with icariin. Icariin and p-icariin also attenuated the changes in oxidative stress and liver injury. We found positive correlations among indices of oxidative stress (malondialdehyde and inducible nitric oxide synthase) and liver injury (alanine aminotransferase, alkaline phosphatase, and lactate dehydrogenase), suggesting that DHV-1 causes significant oxidative damage, which is related to the extent of hepatic injury.

**Conclusions:**

Icariin and p-icariin improved the survival and attenuated oxidative stress and liver dysfunction induced by DHV-1. These outcomes were better in ducklings treated with p-icariin than in those treated by icariin. The clinical effects of both components were related to their antioxidant activities.

## Background

Duck viral hepatitis (DVH) is an acute, contagious, and highly fatal disease of young ducklings [1] that is mainly caused by duck hepatitis virus 1 (DHV-1) belonging to the *Picornaviridae* family [2]. There are currently very few drugs to treat this disease. Some antiviral drugs, especially some traditional Chinese medicines, still need to be comprehensively evaluated before they can be introduced into veterinary medicine, although some compounds are effective in some cases.

DHV-1 infection is often accompanied by severe oxidative stress, which plays a vital role in the pathogenesis of hepatic injury [3,4]. The production of free radicals, such as malondialdehyde (MDA) and nitric oxide (NO), increases oxidative stress and may impair cellular functions, including nucleotide and protein synthesis, thereby contributing to the initiation and progression of hepatic injury [5,6].

Oxidative stress can damage cell components and may cause cell death [7]. Therefore, liver cells possess an effective antioxidant defense system consisting of antioxidant enzymes such as superoxide dismutase (SOD), catalase, and glutathione peroxidase (GSH-Px) to reduce oxidative stress. To date, however, insufficient work has been done to study the role of oxidative damage in the pathogenesis of DVH. Hepatic injury reflects disturbances in liver cell metabolism, which cause characteristic changes in serum enzyme activities. Elevated serum enzymes are indicative of cellular leakage and the loss of functional integrity of affected hepatocytes [8]. Damage to the liver cell plasma membrane causes the release of liver enzymes such as aspartate aminotransferase (AST), alanine aminotransferase (ALT), alkaline phosphatase (ALP), and lactate dehydrogenase (LDH) into blood vessels, while the levels of total protein (TP), albumin, and globulin are decreased in overt hepatitis [9]. Therefore, the serum levels of these markers are useful for assessing the extent and type of hepatocellular damage.

One of the most important properties of flavonoids is their potent antioxidant activity. Flavonoids exert their antioxidant activity by scavenging or quenching free radicals, by chelating metal ions, or by inhibiting the enzymatic systems responsible for producing free radicals [10,11]. Like other flavonoids, epimedium flavones exhibit many biological activities and pharmacological effects, including antioxidant,anti-aging, antitumor, and anti-osteoporosis activities [12-15]. Icariin (C_33_H_40_O_15_, molecular weight: 676.6617) is the main component of epimedium flavones [16]. Because icariin shows poor water solubility, we prepared phosphorylated icariin (p-icariin), which showed significantly increased water solubility compared with native icariin. In our previous study, icariin and p-icariin prevented DHV-1 from invading duck embryonic hepatocytes *in vitro*, with p-icariin showing greater antiviral properties than icariin. To investigate the potential of icariin and p-icariin for preventing DVH, we determined the changes in markers of hepatic injury and oxidative damage, and examined correlations among these markers in the present. To establish an effective clinical treatment for DVH, we also compared the effects of icariin and p-icariin, focusing on their effects on oxidative stress in the pathogenesis of DVH.

## Methods

### Ethics statement

Animal experiments conformed to the Guide for the Care and Use of Laboratory Animals published by the US National Institutes of Health (NIH Publication, Eighth edition, 2011) and was approved by the Nanjing Agricultural University Animal Care Committee. To ameliorate suffering, animals that were not expected to survive were humanely euthanized. All steps were complied with AVMA Guidelines for the Euthanasia of Animals (2013 Edition).

### Reagents and virus

Icariin (lot no. 20121228, net content 89.18%) was bought from Xi’an Grassroot Chemical Engineering Co. Ltd. (Xian, China). Sodium tripolyphosphate (STPP; lot no. 20120706) was bought from Sinopharm Chemical Reagent Co., Ltd. (Shanghai, China). Sodium trimetaphosphate (STMP; lot no. L1226014) was purchased from Aladdin Industrial Corporation (Shanghai, China). Duck SOD (lot no. 201310), GSH-Px (lot no. 201310), catalase (lot no. 201310), MDA (lot no. 201310), and iNOS (lot no. 201310) enzyme-linked immunosorbent assay kits were bought from Biocalvin Company (Suzhou, China). ALT (lot no. 130828), AST (lot no. 130531), ALP (lot no. 130425), LDH (lot no. 130917), TP (lot no. 131031), and albumin (lot no. 130422) assay kits were purchased from AusBio Laboratories Co., Ltd (Shanghai, China). DHV-1 (LD_50_ is 5 × 10^−3^) strain LQ_2_ used in the challenge experiments was supplied by the Shandong Institute of Poultry (Shandong, China, −70°C storage). All of the other chemicals used in this study were of analytical grade.

### Preparation of p-icariin

The STMP-STPP method was used to prepare p-icariin, as follows. First, 2.5 g STMP and 1.0 g STPP were mixed in 50 mL of distilled water, with stirring. Icariin (500 mg) was dissolved in 100 mL of distilled water and added to the STMP-STPP reagent, and stirred in a water bath at 70°C for 6 h at pH 9. The resulting solution was dialyzed, purified by sevage method and column chromatography of Sephadex G-200 (2 cm × 100 cm) and then eluted with distilled water, and lyophilized to yield p-icariin. The icariin and phosphate contents were 56.41% and 34.85%, as determined by the icariin standard method and the molybdenum blue colorimetric method, respectively. The Fourier transform–infrared spectra of icariin and p-icariin were recorded using a Nicolet 200 Magna-IR spectrometer (Nicolet Instrument Corp., Madison, WI, USA). The pre-modification and post-modification waveforms were basically identical, indicating that icariin and p-icariin have similar structures. Specific absorption peaks at 3200–3650, 1651.00, 1600.97, 1500.90, and 1453.81 cm^−1^ were found for icariin and p-icariin. p-icariin also displayed absorption peaks at 1160.61 and 918.15 cm^−1^, corresponding to the phosphate group. These findings confirmed that the SMTP-STPP method successfully yield icariin.

### Animals and treatments

One-day-old cherry valley ducks were purchased from Nanjing Tangquan Poultry Farm (Nanjing, China). Ducks were housed in wire cages (60 cm × 100 cm) in air-conditioned rooms at 37°C with lights on for 24 h before the study. The temperature was gradually reduced to room temperature and 12-h light/12-h dark phases, which were kept constant for the remainder of the study. Ducks were fed a commercial starter diet provided by the feed factory of Jiangsu Academy of Agricultural Science (Nanjing, China).

Three-day-old cherry valley ducks (n = 180) were randomly divided into four groups: icariin-treated group, p-icariin-treated group, virus control (VC) group, and a blank control (BC) group (separately reared). Ducklings allocated to the icariin, p-icariin, and VC groups were intramuscularly injected with 0.2 mL of DHV-1 (20 × LD_50_) per duckling. Ducklings allocated to the icariin or p-icariin groups were given aqueous icariin or p-icariin at the dosage of 31.25 mg/Kg of per duckling, *SID*, for 3 days starting on the same day as DHV-1 injection. Before used, icariin was prepared with 3% ethanol dissolved, and added a co-solvent 0.5% Tween 80. In order to ensure the consistency of test, we added the equal amount of ethanol and Tween 80 in the drinking in the p-icariin, VC and BC groups. The dissolution characteristic of p-icariin is instant into the water. Blood samples were taken from five ducklings in each group at 4, 8, and 54 h after injecting DHV-1. Half of each blood sample was treated with heparin for anticoagulation and the remainder was left to coagulate. Number of ducks which had been taken blood samples needed to eliminate (n = 15 per group).

The clinical symptoms and number of dead ducklings were monitored daily, continuous observation for 7 days. The dead ducklings were promptly autopsied for pathological assessment to exclude any ducklings without pathological changes (petechial hemorrhage in liver, convulsion, opisthotonos). The time of death was recorded for each duckling and the survival rate was recorded until no death was found.

### Indices of oxidative stress and liver injury

The plasma SOD, GSH-Px, catalase, iNOS, and MDA levels at 8 and 54 h after DHV-1 injection were measured using duck SOD, GSH-Px, catalase, iNOS, and MDA assay kits, respectively.

The serum ALT, AST, ALP, LDH, TP, and albumin levels at 4, 8, and 54 h after DHV-1 injection were determined using enzymatic colorimetric assay kits on an automatic biochemistry analyzer (Hitachi 7180 Automatic Analyzer; Hitachi, Japan) at Nanjing Shihuang Institute of Animal Science and Technology. The serum globulin level was calculated as the serum TP level minus the serum albumin level.

### Statistical analysis

Correlations among markers of oxidative stress and liver injury were determined using Pearson’s correlation coefficient. Comparisons among the experimental groups were made using one-way analysis of variance and differences between specific groups were determined using Duncan’s Multiple Range Test. Results are expressed as means ± standard error for five ducklings in each group. The difference in the survival rate was determined using the *χ*^2^ test. Differences were considered statistically significant at *p* < 0.05. All of the statistical analyses were conducted using SPSS Software Package version 20.0 (IBM, Armonk, NY, USA).

## Results

Effects of icariin and p-icariin on liver pathology and the survival rate of ducklings exposed to DHV-1.

**Figure 1:**
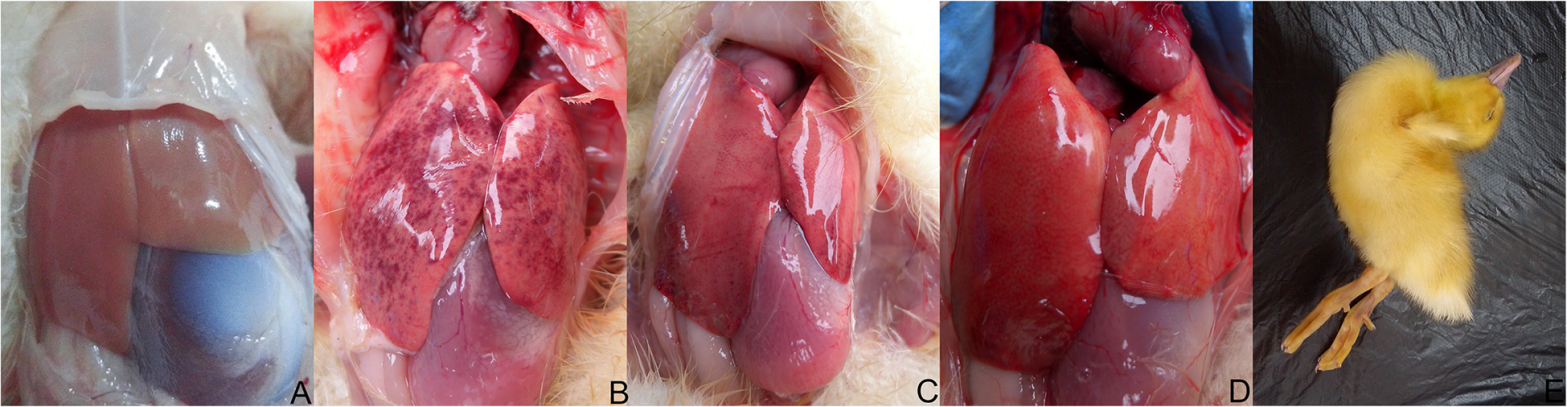
**Visual assessment of liver pathology. (A)** The livers of ducklings in the BC group are normal without pathological changes. **(B)** Extensive pathological changes can be seen in ducklings in the VC group, including fading of the liver color, swelling, and scattered hemorrhage. **(E)** Overt DVH is associated with opisthotonus. **(C, D)** Administration of icariin **(C)** or p-icariin **(D)** alleviates the pathological changes induced by DHV-1. The color of the livers in both groups is similar to those of the BC group. The extent of swelling is reduced and there are fewer hemorrhages in both groups, especially in the p-icariin group.

The pathological changes in the liver are shown in Figure 1. As shown in Table 1, the survival rates in the BC, VC, icariin, and p-icariin groups were 100%, 0%, 20% and 30%, respectively. All of the ducklings in the VC group died within 96 h of exposure to DHV-1. The survival rate in the p-icariin group was significantly greater than that in the VC group (*p* < 0.05). However, the survival rate was not significantly different between the icariin and p-icariin groups.

**Table 1:** **Survival rate**

**Group**	**Number of ducklings**	**Number of survival ducklings**	**Survival rate (%)**
Icariin	30	6	20.0^bc^
P-icariin	30	9	30.0^b^
VC	30	0	0.0^c^
BC	30	30	100.0^a^

^a–c^Values within a column without the same superscripts (^a–c^) are significantly different (*p* < 0.05). ^bc^ with ^b^ and ^c^ were no significant differences.

### Indices of oxidative stress

**Figure 2:**
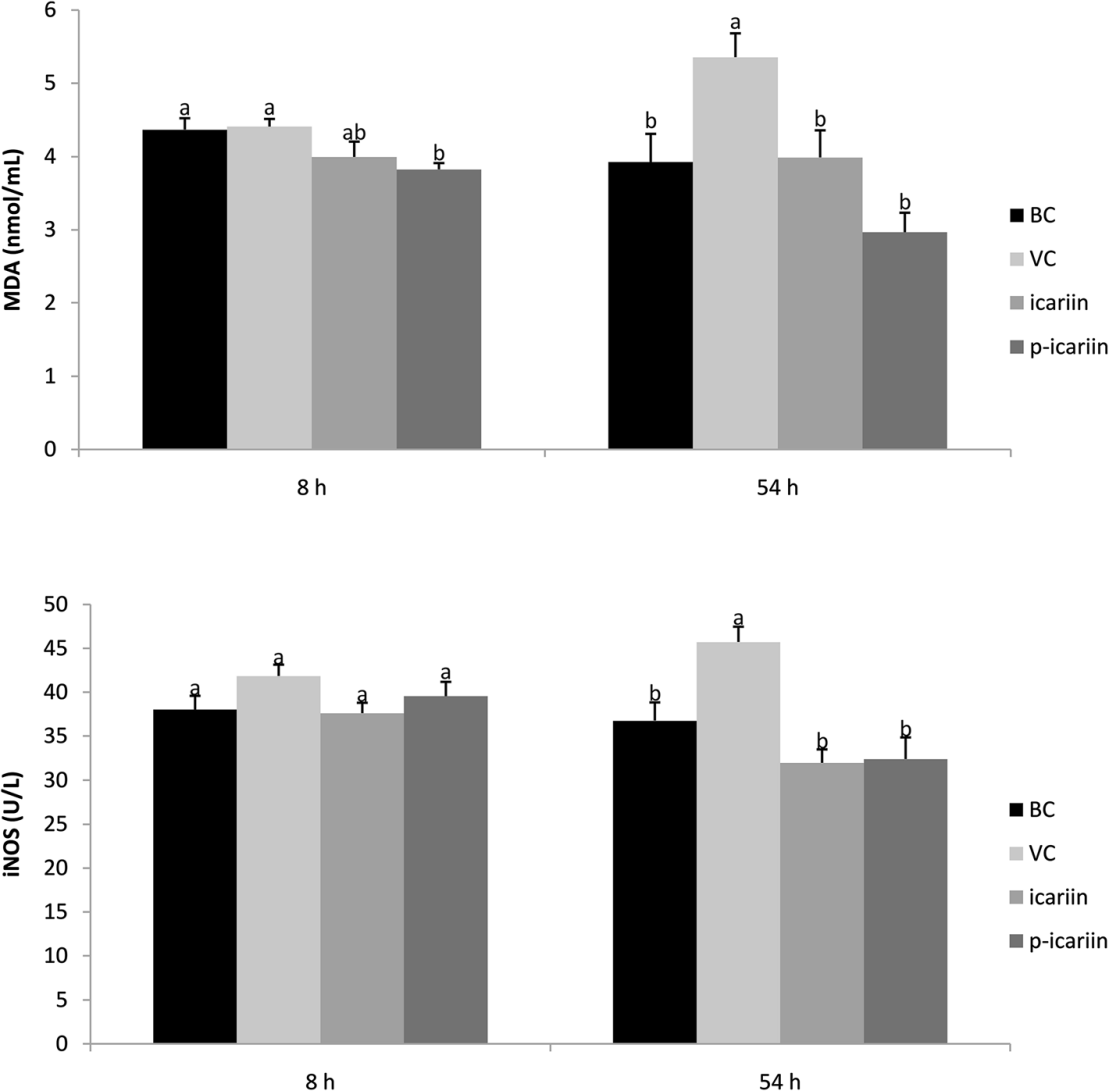
**Plasma iNOS and MDA levels.** ^a,b^Bars without the same superscripts (^a,b^) at the same time are significantly different (*p* < 0.05). ^ab^ with ^a^ and ^b^ were no significant differences.

Figure 2 shows the plasma iNOS and MDA levels in each group at 8 and 54 h after exposure to DHV-1. The plasma iNOS level was not significantly different between each group. However, the MDA level was lower in the p-icariin group than in the VC and BC groups at 8 h. At 54 h, the iNOS and MDA levels were markedly higher in the VC group than in the other groups, but they were not significant different between the BC, icariin, and p-icariin groups.

**Figure 3:**
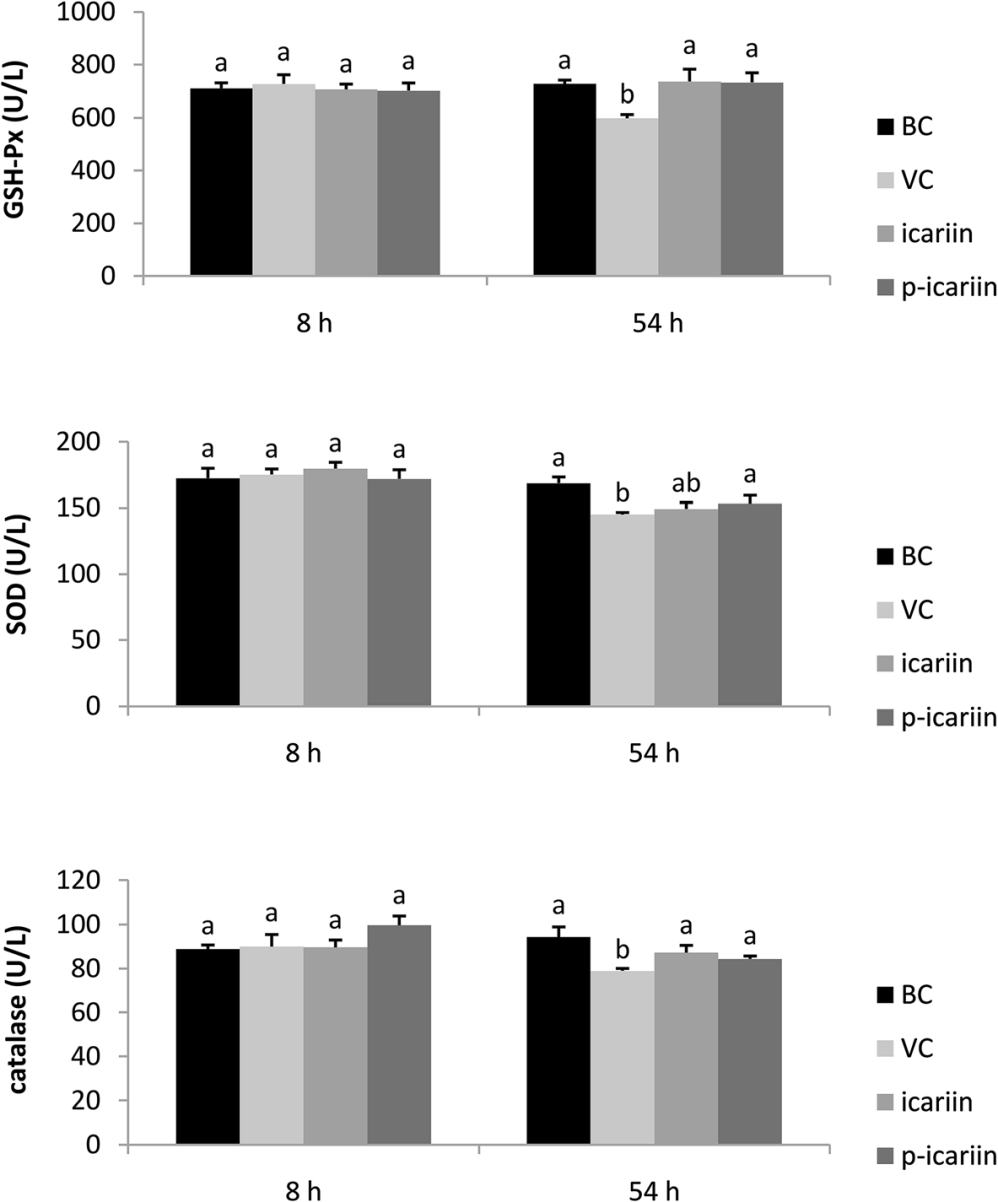
**Plasma GSH-Px, SOD, and catalase levels.** ^a,b^Bars without the same superscripts (^a,b^) at the same time are significantly different (*p* < 0.05). ^ab^ with ^a^ and ^b^ were no significant differences.

Figure 3 shows the plasma GSH-Px, SOD, and catalase levels in each group at 8 and 54 h. Plasma GSH-Px, SOD, and catalase levels were not significantly different among the four groups at 8 h. At 54 h, GSH-Px and catalase levels were significantly greater in the BC, icariin, and p-icariin groups than in the VC group, but were not significant different among the BC, icariin, and p-icariin groups. SOD level at 54 h was significantly greater in the BC and p-icariin groups than in the VC group, and was slightly greater in the icariin group than in the VC group.

### Indices of liver injury

Table 2 shows the indices of liver injury in each group in the early (4 and 8 h) and the late (54 h) stages of infection. The serum ALT levels were consistently greater in the VC group than in the other groups at all times. At 4 and 8 h, there were no significant differences in ALT levels among the icariin, p-icariin, and BC groups. At 54 h, ALT levels were significantly greater in the icariin and p-icariin groups than in the BC group, but were not significantly different between the icariin and p-icariin groups.

**Table 2:** **Indices of liver injury**

		**Group**			
	**Time (h)**	**BC**	**VC**	**Icariin**	**P-icariin**
	4	12.000 ± 3.512^b^	31.800 ± 3.987^a^	15.000 ± 4.000^b^	17.000 ± 1.414^b^
ALT (IU/L)	8	12.000 ± 1.000^b^	24.750 ± 1.109^a^	19.750 ± 4.922^b^	18.500 ± 5.500^b^
	54	9.400 ± 2.112^c^	49.200 ± 3.264^a^	33.500 ± 0.500^b^	30.333 ± 0.333^b^
	4	23.750 ± 1.315^b^	30.750 ± 3.816^b^	49.800 ± 1.600^a^	44.700 ± 2.563^a^
AST (IU/L)	8	24.750 ± 2.496^b^	27.000 ± 1.577^b^	48.600 ± 7.800^a^	51.600 ± 6.158^a^
	54	25.000 ± 6.245^b^	41.330 ± 4.667^a^	51.000 ± 6.557^a^	52.667 ± 8.930^a^
	4	23.220 ± 1.458^a^	17.220 ± 2.449^b^	24.400 ± 1.203^a^	21.670 ± 1.564^ab^
TP (g/L)	8	20.740 ± 0.627^a^	17.060 ± 0.848^b^	17.920 ± 1.498^a^	20.440 ± 1.608^a^
	54	23.380 ± 0.711^a^	15.820 ± 1.639^b^	22.410 ± 1.559^a^	19.100 ± 1.774^ab^
	4	9.675 ± 0.731^a^	7.987 ± 0.067^a^	8.976 ± 0.635^a^	9.090 ± 0.270^a^
Albumin (g/L)	8	8.833 ± 0.318^a^	6.675 ± 0.217^b^	6.360 ± 0.386^b^	7.056 ± 0.851^ab^
	54	8.760 ± 0.328^a^	5.267 ± 0.260^c^	7.680 ± 0.705^ab^	6.720 ± 0.413^b^
	4	13.350 ± 0.235^a^	12.200 ± 0.769^a^	15.432 ± 0.574^a^	13.464 ± 0.702^ab^
Globulin (g/L)	8	12.460 ± 0.304^a^	13.080 ± 0.761^a^	12.240 ± 1.043^a^	13.392 ± 0.763^a^
	54	14.620 ± 0.461^a^	11.300 ± 0.551^c^	13.848 ± 0.794^ab^	12.480 ± 0.317^b^
	4	695.750 ± 35.172^a^	713.250 ± 44.618^a^	618.720 ± 51.188^a^	612.480 ± 51.006^a^
ALP (IU/L)	8	613.000 ± 32.929^a^	640.250 ± 18.400^a^	624.160 ± 66.946^a^	657.280 ± 65.141^a^
	54	659.000 ± 17.459^b^	830.000 ± 41.540^a^	651.400 ± 82.500^b^	630.600 ± 38.259^b^
	4	786.000 ± 61.093^a^	849.000 ± 78.945^a^	926.000 ± 36.865^a^	814.200 ± 54.631^a^
LDH (IU/L)	8	561.000 ± 20.228^a^	668.250 ± 29.565^a^	692.200 ± 54.310^a^	684.800 ± 50.258^a^
	54	613.400 ± 23.359^c^	1024.66 ± 57.704^a^	831.333 ± 67.814^b^	758.500 ± 99.500^bc^

^a-c^Values within the same row without the same superscripts (^a-c^) are significantly different (*p* < 0.05). ^ab^ with ^a^ and ^b ^were no significant differences. ^bc^ with ^b^ and ^c^ were no significant differences.

The serum AST levels in the VC group tended to be greater than those in the BC group at all times. AST levels were significantly greater in the icariin and p-icariin groups than in the BC and VC groups at 4 and 8 h. At 54 h, serum AST levels were similar among the BC, VC, and icariin groups, and were significantly greater in the p-icariin group than in the BC group.

The serum ALP levels at 4 and 8 h were similar among the four groups. However, at 54 h, serum ALP levels were markedly lower in the icariin, p-icariin, and BC groups than in the VC group, and were not significantly different among the icariin, p-icariin, and BC groups at this time.

The serum LDH levels at 4 and 8 h were not significantly different among the four groups. At 54 h, serum LDH levels were significantly lower in the BC, icariin, and p-icariin groups than in the VC group. Serum LDH tended to be greater in the p-icariin group than in the BC group, although the difference was not significant.

The serum TP levels were significantly greater in the BC and icariin groups than in the VC group at all sampling times. The serum TP levels were greater in the p-icariin group than in the VC group at 4 and 54 h, although the differences were not significant. At 8 h, the serum TP level was greater in the p-icariin group than in the VC group.

The serum albumin levels in the icariin and p-icariin groups were not significantly different from those in the VC group at 4 or 8 h. However, serum albumin levels at 8 h were significantly lower in the VC and icariin groups than in the BC group. At 54 h, serum albumin levels were significantly greater in the icariin, p-icariin, and BC groups than in the VC group, and were lower in the p-icariin group than in the BC group.

At 4 and 8 h, serum globulin levels were not significantly different among the four groups. However, at 54 h, serum globulin levels were greater in the icariin and p-icariin groups than in the VC group and were significantly lower in the p-icariin group than in the BC group.

### Correlations among indices of liver injury and oxidative stress

The correlation coefficients among indices of liver injury and oxidative stress measured at 54 h are listed in Table 3. As shown in this table, ALT, ALP, and LDH were positively correlated with MDA and iNOS. TP, albumin, and globulin were positively correlated with catalase, SOD, and GSH-Px. ALT, AST, ALP, and LDH were negatively correlated with catalase, SOD and GSH-Px, while AST, TP, albumin, and globulin were negatively correlated with MDA and iNOS. ALT was significantly and negatively correlated with SOD, catalase, and albumin (all *p* < 0.05). GSH-Px was significantly and negatively correlated with iNOS (*p* < 0.05), MDA (*p* < 0.05) and LDH (*p* < 0.01). SOD was significantly and negatively correlated with LDH (*p* < 0.05).

**Table 3:** **Pearson’s correlation coefficients between indices of liver injury and oxidative stress**

	**GSH-Px**	**iNOS**	**Catalase**	**SOD**	**MDA**	**ALT**	**AST**	**TP**	**Albumin**	**Globulin**	**ALP**	**LDH**
GSH-Px	1	−0.775^*^	0.510	0.398	−0.773^*^	−0.637	−0.048	0.759^*^	0.631	0.697	−0.401	−0.708^*^
iNOS		1	−0.137	0.112	0.826^*^	0.279	−0.357	−0.573	−0.487	−0.487	0.286	0.290
Catalase			1	0.691	−0.370	−0.780^*^	−0.098	0.605	0.501	0.680	−0.325	−0.705
SOD				1	−0.140	−0.805^*^	−0.380	0.212	0.320	0.217	−0.488	−0.821^*^
MDA					1	0.409	−0.397	−0.420	−0.315	−0.424	0.419	0.436
ALT						1	0.445	−0.642	−0.779^*^	−0.574	0.256	0.966^**^
AST							1	−0.310	−0.501	−0.202	−0.435	0.479
TP								1	0.871^**^	0.943^**^	0.084	−0.604
Albumin									1	0.696	0.054	−0.749^*^
Globulin										1	0.149	−0.497
ALP											1	0.362
LDH												1

*p < 0.05.

**p < 0.01.

## Discussion

This study showed that administration of icariin and p-icariin significantly improved the survival rate of ducklings exposed to DHV-1, as illustrated in Table 1, demonstrating the curative potential of icariin and p-icariin. Administration of icariin or p-icariin also alleviated visible signs of liver injury. The higher survival rate in the p-icariin group than in the icariin group suggests that p-icariin might be a better treatment of DHV-1.

Oxidative stress is attributed to an imbalance between reactive oxygen species and the antioxidant defense mechanisms of a cell or tissue, causing lipid peroxidation, DNA damage, and the inactivation of many enzymes [17]. Lipid peroxidation by free radicals leads to the oxidative destruction of polyunsaturated fatty acids that form the cellular membranes. The destruction of polyunsaturated fatty acids results in the production of toxic and reactive aldehyde metabolites such as MDA [18]. Therefore, the MDA content reflects the extent of lipid peroxidation and is an indirect marker for the extent of hepatocyte injury *in vivo* [19]. In addition, NO and superoxide radicals interact to form peroxinitrite, which is an important mediator of free radical toxicity [6]. iNOS is expressed in hepatocytes and macrophages and produces abundant NO in response to cytokines, a process that may exacerbate lipid peroxidation-induced hepatocyte injury. It was reported that viral infection increases iNOS mRNA expression and NO production. In turn, NO promotes viral replication in infected cells [20-23]. In our study, at 54 h after exposure to DHV-1, iNOS and MDA levels were significantly greater in the VC group than in BC group. These changes were associated with increased severity of liver disease. Their levels were significantly reduced in ducklings treated with icariin and p-icariin groups, which indicates that both components are capable of attenuating peroxidation-related injury (Figure 2).

The enzymatic antioxidant defense system serves to protect against lipid peroxidation. The main enzymes in this system are SOD, catalase, and GSH-Px, which act cooperatively or synergistically to protect cells from oxidative stress. However, optimal protection is only achieved when the activities of these enzymes reach an appropriate balance [24]. At 8 h after exposure to DHV-1, the plasma GSH-Px, SOD, and catalase levels were not significantly different among the four groups, which indicates that the free radical levels were balanced with the enzyme levels during the initial stage of infection. However, by 54 h, the levels of these enzymes were significantly lower in the VC group than in the BC group. SOD protects against superoxide radicals, which damage the cell membrane and its integrity. Catalase primarily decomposes H_2_O_2_ to H_2_O, like GSH-Px, but at a much faster rate [25]. GSH-Px may also play an important role in the removal of lipid hydroperoxides [26]. The balance between these enzymes is important for the efficient removal of oxygen radicals from tissues [27]. Therefore, a reduction in the enzyme levels of one or more of these enzymes may have deleterious effects following the accumulation of superoxide radicals and H_2_O_2_. Notably, we found significant increases in the levels of these enzymes in the icariin and p-icariin groups relative to the VC group, and the levels were similar to those in the BC group (Figure 3). These results indicate that icariin and p-icariin attenuate oxidative stress following exposure to DHV-1, and highlight the potential for reducing liver injury during treatment of viral infection. The significant negative correlations between indices of liver injury (i.e. ALT and LDH) and indices of oxidative stress (i.e. SOD, catalase, and GSH-Px) also support these results. Intriguingly, there were no significant differences in the antioxidant properties of phosphorylated and native icariin.

Serum ALT, AST, ALP, and LDH levels are widely used to evaluate the necroinflammatory activity in liver diseases [28-30] because they are positively correlated with the extent of hepatocytes damage. In our study, we found that the serum ALT, AST, ALP, and LDH levels were significantly greater in the VC group than in the BC in the late stage of infection (i.e., at 54 h), which indicates that DHV-1 caused severe liver injury. Serum ALT levels were lower in the icariin and p-icariin groups than in the VC group and were similar to those in the BC group. These results indicated that icariin and p-icariin attenuate liver injury, reducing the release of ALT into blood. The modest increases of serum AST in the VC group in the early stage of infection indicate that hepatocytes, or at least mitochondria, were not severely damaged, although DHV-1 did reduce the integrity of the hepatocytes (Table 2). The serum AST levels were significantly higher in the icariin and p-icariin groups than in the BC group. This phenomenon might be related to hepatocyte apoptosis or fragmentation caused by icariin and p-icariin in the initial stage of DVH, and the inhibition of DHV-1 replication *in vivo*. The significant increases in serum ALP and LDH levels are consistent with severe hepatocyte injury in the VC group, but the decreases observed in the icariin and p-icariin groups were not clinically significant. At 54 h, the serum ALP and LDH levels were significantly greater in the VC group than in other three groups, which was indicatived of severe liver injury in the late stage of infection in the VC group. Serum ALP and LDH did not increase significantly before 8 h in any of the groups. These results imply that, in the early stage of infection, DHV-1 was still replicating and its proliferation was too low to cause extensive hepatocyte injury. These results are consistent with those of our previous study [31].

We found that indices of liver injury (ALT, ALP, and LDH) were positively correlated with markers of oxidative stress (MDA and iNOS) and negatively correlated with antioxidant enzymes (GSH-Px, SOD, and catalase) (Table 3). In the late stage of infection, it seems that oxidative stress caused severe damage to hepatocytes and aggravated the severity of DVH in the VC group. Thus, the serum ALT, ALP, and LDH levels increased in the VC group. Icariin and p-icariin appeared to balance the free radical levels and significantly reduced the serum ALT, ALP, and LDH levels in ducklings exposed to DHV-1. These results illustrate the hepatoprotective effects of icariin and p-icariin are mediated by their antioxidative effects. The serum ALT, ALP, and LDH levels in the p-icariin group were lower than those in the icariin group, although the differences were not significant. These findings might also be related to the slightly higher survival rate in the p-icariin group than the icariin group. SOD was significantly and negatively correlated with ALT and LDH (*p* < 0.05), which suggests that SOD is one of the most important antioxidant markers in DVH. Notable, p-icariin had greater effects on SOD, ALT, and LDH than icariin, which is consistent with the greater survival rate in the p-icariin group (Table 1). In the first 8 h, the serum albumin level was significantly lower in the VC group than in the BC group, which was probably due to a reduction in the hepatocytes’ ability to synthesize albumin following infection with DHV-1 (Table 2). Administration of icariin or p-icariin groups increased the albumin level compared with the VC group at 54 h after infection, but the levels in both groups were still lower than those in the BC group. These improvements in albumin levels might be related to the inhibitory effects of icariin and p-icariin against DHV-1. Both components may inhibit the replication of DHV-1, and ultimately inhibiting the protein synthesis of DHV-1 in hepatocytes. In the early stage of infection, there was no significant difference in serum globulin levels among the four groups while the TP levels followed a similar pattern to albumin levels (Table 2). In the later stage of infection, abundant free radicals probably caused serious liver injury in the VC group. Free radicals also affect proteins and we found positive correlations between markers of protein damage and oxidative stress in this paper (Table 3). Accordingly, the serum TP, albumin, and globulin levels in the VC group were significantly lower than those in the BC group. Globulin has some immunologic roles. Thus, the lower resistance to DHV-1 in the VC group may be related to a decrease in globulin levels, and potentially aggravated the severity of DVH. Icariin and p-icariin can scavenge free radicals, reducing the extent of hepatocyte damage. Therefore, the serum TP, albumin, and globulin levels of icariin and p-icariin groups declined modestly or remained about the same in these two groups. Serum MDA, GSH-Px, SOD, catalase, and iNOS levels are related to the abundance of free radicals while the serum ALT, AST, ALP, LDH, TP, albumin, and globulin levels are related to the severity of DVH. These correlations are particularly important because they highlight the critical role of free radicals in the pathogenesis of DVH (Table 3). The anti-DVH effects of icariin and p-icariin are probably related to their free radical scavenging properties. Although the free radical scavenging properties appear to be similar between icariin and p-icariin, p-icariin seemed to have slightly greater anti-DVH effects than icariin, although the differences were not statistically significant. Overall, these findings indicate that the clinical effects of icariin and p-icariin are not simply related to their antioxidant properties. Further studies are needed to investigate the other relevant mechanisms by which icariin and p-icariin protect against DVH.

## Conclusion

Infection with DHV-1 caused severe oxidative stress and liver injury in ducklings. We found that several indices of liver injury (ALT and LDH) were significantly and negatively correlated with indices of antioxidant capacity (GSH-Px, catalase, and SOD), and that SOD might be one of the most important factors for limiting the degree of liver injury. Icariin and p-icariin possess antioxidant activities and are hepatoprotective, as demonstrated by the reductions in serum liver injury markers and increased in antioxidative enzyme levels. These changes could alleviate liver injury, and are at least partly related to antioxidant properties of both components. Although, both components had similar free radical scavenging effects, p-icariin exerted greater hepatoprotective effects than icariin.
